# How Changes in Psychosocial Job Characteristics Impact Burnout in Nurses: A Longitudinal Analysis

**DOI:** 10.3389/fpsyg.2016.01082

**Published:** 2016-07-26

**Authors:** Renato Pisanti, Margot van der Doef, Stan Maes, Laurenz Linus Meier, David Lazzari, Cristiano Violani

**Affiliations:** ^1^Faculty of Psychology, Niccolò Cusano UniversityRome, Italy; ^2^Health, Medical and Neuropsychology Unit, Institute of Psychology, Leiden UniversityLeiden, Netherlands; ^3^Institute of Work and Organizational Psychology, University of NeuchatelNeuchatel, Switzerland; ^4^Section of Clinical and Medical Psychology, Hospital S. MariaTerni, Italy; ^5^Department of Psychology, Sapienza University of RomeRome, Italy

**Keywords:** burnout, nurses, job demands control support model, longitudinal study, change model

## Abstract

**Aims:** The main aim of this longitudinal study was to test the Job Demand-Control-Support (JDCS) model and to analyze whether changes in psychosocial job characteristics are related to (changes in) burnout.

**Background:** Previous studies on the effects of JDCS variables on burnout dimensions have indicated that the iso-strain hypothesis (i.e., high job demands, low control, and low support additively predict high stress reactions) and the buffer hypotheses (i.e., high job control and/or social support is expected to moderate the negative impact of high demands on stress reactions) have hardly been examined concurrently in a longitudinal design; and that the effects of changes of psychosocial job variables on burnout dimensions have hardly been analyzed.

**Design:** This two wave study was carried out over a period of 14 months in a sample of 217 Italian nurses.

**Method:** Hierarchical regression analyses were used to test the cross lagged main and interactive effects of JDCS variables, and to analyse the across-time effects of changes in JDCS dimensions on burnout variables.

**Results:** The Time 1 job characteristics explained 2–8% of the variance in the Time 2 burnout dimensions, but no support for the additive, or the buffer hypothesis of the JDCS model was found. Changes in job characteristics explained an additional 3–20% of variance in the Time 2 burnout dimensions. Specifically, high levels of emotional exhaustion at Time 2 were explained by high levels of social support at Time 1, and unfavorable changes in demands, control, and support over time; high depersonalization at Time 2 was explained by high social support at time 1 and by an increase in demands over time; and high personal accomplishment at Time 2 was predicted by high demands, high control, interactive effect demands × control × social support, at Time 1, and by a decrease in demands over time. No reversed effects of burnout on work characteristics have been found.

**Conclusion:** Our findings suggest that the work environment is subject to changes: the majority of employees experienced considerable changes in all job conditions over time. These changes impacted employee burnout. Limitations and implications of the study are discussed.

## Introduction

A number of studies have shown that nurses, in the course of their careers, experience a great deal of stress that may have implications for their physical, and mental health status (McVicar, 2003; Cortese et al., 2010; Pisanti et al., 2011, 2015; Van Bogaert et al., 2014; Converso et al., 2015; Panagopoulou et al., 2015; Welp et al., 2015; Giorgi et al., 2016).

One of the most-researched long-term consequence of stress in nurses is burnout, which is defined as a multidimensional construct with three facets: emotional exhaustion, depersonalization, and lack of personal accomplishment (Maslach et al., 2001). Emotional exhaustion refers to feelings of being emotionally overextended and exhausted by one's work and contact with other people. Depersonalization refers to an unfeeling and impersonal response toward the recipients of one's care or service. Lack of personal accomplishment refers to a decline in one's feelings of competence and successful achievement in one's work. Burnout prevalence among nurses varies between 2 and 11% (Bourbonnais et al., 1999; Kilfedder et al., 2001; Schaufeli, 2007). Over the past decade its prevalence has increased substantially. The current economic crisis in Italy and other countries now forces nurses to work longer hours for lower pay, and there is also less job stability (Van Bogaert et al., 2009; Renzi et al., 2012; Canadas-De la Fuente et al., 2015). More recent studies have found higher percentages of nurses with high and moderate levels of burnout. For example, a study of Renzi et al. (2012) found that 46% of the nurses in their sample had elevated scores on emotional exhaustion.

Previous studies on the effects of psychosocial job variables on burnout dimensions have indicated that the iso-strain hypothesis (i.e., high job demands, low control, and low support *additively* predict high stress reactions) and the buffer hypotheses (i.e., high job control and/or social support is expected to moderate the negative impact of high demands on stress reactions) have hardly been examined concurrently in a longitudinal design. Moreover, most previous studies used a static approach focusing on the absolute level of psychosocial job characteristics at a given time point, neglecting that work conditions are likely to change (Roe, 2008; Schaufeli et al., 2009; Tang, 2014). To consider the dynamic nature of work conditions and its effects on well-being, we examined whether changes in work conditions have an unique effect on burnout dimensions that go above and beyond the static levels of work conditions.

Therefore, the main goal of this longitudinal study was to test the Job Demand-Control-Support (JDCS) model and to analyze whether changes in psychosocial job characteristics are related to (changes in) burnout.

### The job demands control social support model

Psychosocial job characteristics may contribute to the incidence of burnout among health care employees (Schaufeli, 2007). Job demands, job control (skill discretion and decision authority), and social support from colleagues and supervisor are the core dimensions of the JDCS model (Johnson and Hall, 1988; Karasek and Theorell, 1990). The basic assumption of this model states that high job demands, low control, and low support *additively* predict high stress reactions (iso-strain hypothesis). On the one hand, researchers have focused on the buffer hypothesis, stating that high job control, and/or social support is expected to moderate the negative impact of high demands on stress reactions (Karasek and Theorell, 1990). This theoretical issue has an important implication for job redesign. A *buffer* effect of control and social support would lead to recommendations to increase job control and social support in order to decrease the detrimental effects of demands. On the other hand, if the “iso-strain” hypothesis is valid and poor well-being is the result of additive effects of demands, control, and social support, it would be insufficient to focus solely on the increment of job control and social support, with the high demands maintaining their unfavorable effect on employees health.

Several authors (van der Doef and Maes, 1999; de Lange et al., 2003; Häusser et al., 2010) reviewed the main assumptions of the JDCS model. Overall, a general conclusion from these reviews is that the additive hypotheses were most investigated and received more support than the buffer hypotheses.

Several studies have examined the main effects of the JDCS variables on burnout dimensions. These studies suggest that job demands (such as time pressure and workload) are a stronger predictor of emotional exhaustion and depersonalization than control but a weaker predictor of personal accomplishment than control (Lee and Ashforth, 1996; Schaufeli, 2007). Social support appears to be associated with each burnout dimension, although the relationship is less strong than in the case of job demands (Schaufeli, 2007). These findings were also confirmed in nursing populations (e.g., Proost et al., 2004; Bakker et al., 2005; Hochwalder, 2006, 2007). Reviewing the studies on nurses, Pisanti et al. (2012) found that the strain hypothesis was more frequently investigated than the buffer hypothesis: whereas 22 studies examined the additive effects of job demands and control only seven studies examined the interaction between these two psychosocial dimensions. Additive effects of demands and control were found in 7 of the 22 studies that tested this hypothesis, whereas only the study of De Rijk et al. (1998) found a buffer effect under condition of a third variable, i.e., active coping. In this study, control moderated the negative effects of job demands on emotional exhaustion only in the subsample of nurses that showed higher values on active coping. In addition, the iso-strain hypothesis has been supported by the findings of three studies (Bourbonnais et al., 1999; Hochwalder, 2006, 2007) out of 12 that tested this hypothesis, whereas only one study (Proost et al., 2004) tested the three way interaction and found support only for personal accomplishment. Moreover, Pisanti et al. (2012) found that emotional exhaustion was the most frequently investigated burnout dimension (in e.g., Landsbergis, 1988; Bourbonnais et al., 1998, 1999; de Jonge et al., 1999; Tummers et al., 2002; Proost et al., 2004; Bakker et al., 2005; Schmidt and Diestel, 2010). Finally, two longitudinal studies (Bourbonnais et al., 1999; Gelsema et al., 2006) on nurses failed to support both hypotheses.

Although the longitudinal research designs are more suitable to draw conclusions concerning the causal relations among the study concepts than cross-sectional designs, we should acknowledge that the vast majority of existing longitudinal studies on job stress, and occupational strain focused on the influence of occupational stressors on a stress reaction at a later point in time (Taris and Kompier, 2003). For instance, a study among nurses (Ganster et al., 2001) found that, after controlling for the dependent variable at Time 1, neither the main effects of job demands, and control, nor their interactive term, accounted for significant portions of explained variance in mental health after 5 years. However, as suggested by several authors (e.g., de Lange et al., 2002; Roe, 2008; Boersma and Lindblom, 2009; Melamed et al., 2011; Schaufeli et al., 2011) the work environment is not a static phenomenon, it is dynamic, and susceptible to change. This is also shown by weak autocorrelations found in studies that analyzed the (normative) stability both of psychosocial job dimensions (e.g., Gelsema et al., 2006; Schaufeli et al., 2009; Adriaenssens et al., 2013) and burnout variables (e.g., Burisch, 2002; Rudman and Gustavsson, 2012).

A limited number of studies examine the influence of changes of psychosocial job dimensions on burnout outcomes. For example, in a longitudinal research with a 1–year time interval conducted among 201 Dutch telecom managers, Schaufeli et al. (2009) found that increases in job demands (i.e., overload, emotional demands, and work-home interference) and decreases in job resources (i.e., social support, autonomy, opportunities to learn, and feedback) were associated with increases of emotional exhaustion and cynicism over time. Likewise, Bourbonnais et al. (1999), in a sample of Canadian nurses, examined changes in the dimensions of the demand-control model, and found significant main effects of adverse changes in job strain condition (high demands and low control) across time, on emotional exhaustion over time. Finally, Gelsema et al. (2006), in a sample of nurses, found that an increase in job demands (i.e., workload and physical demands) was associated with increases in emotional exhaustion across time. In this latter study, the authors measured psychosocial job variables through an occupation specific measure.

Some authors (Kasl, 1996; Narayanan et al., 1999; van der Doef and Maes, 1999) have argued that generic measures to assess occupational stressors and resources might not adequately reflect the specific workplace conditions, and have pointed out the need for more occupation-specific assessment. They suggest that occupation-specific measurement of demands, control, and support could improve the explanatory and predictive power of the JDCS model (Kasl, 1996; van der Doef and Maes, 2002). Therefore, in the present study a measure to assess specifically nurse's job characteristics was used.

To recapitulate, the previous studies on the effects of JDCS variables on burnout have indicated four issues that we will deal with in the present research: (a) most of studies have examined the hypotheses of the JDCS model on emotional exhaustion, whereas the other two dimensions, depersonalization, and personal accomplishment, have been studied less frequently, (b) the iso-strain hypothesis and the buffer hypotheses have hardly been examined concurrently in a longitudinal design, (c) the effects of changes of psychosocial job variables on burnout dimensions are hardly examined, and (d) it would be advisable to adopt occupation-specific measures to examine the effects postulated by the JDCS model.

### Research hypotheses

On the basis of the theory and empirical studies described earlier, two hypotheses are addressed in this longitudinal study.

The first hypothesis deals with the prospective effects of the JDCS dimensions on burnout. After controlling for the effects of each Time 1 burnout dimension and demographic variables (age and gender), high job demands, low job control, and low social support at Time 1 will be additively associated with high levels of burnout at Time 2 (high scores of emotional exhaustion and depersonalization, low scores of personal accomplishment; *Hypothesis 1a*). Furthermore, in line with the JDCS model (Karasek and Theorell, 1990) the three-way interaction job demands, job control, and social support will explain a significant proportion of the variance in burnout. This interaction will indicate that high job control combined with high social support will buffer the impact of job demands on burnout (*Hypothesis 1b*).

The second hypothesis is concerned with the prospective effects of *changes* in JDCS variables on burnout. More specifically, we propose that increases in job demands and decreases in job control and social support (from Time 1 to Time 2) are associated with increases in emotional exhaustion and depersonalization and decreases in personal accomplishment (*Hypothesis 2*).

## Methods

### Sample and procedure

In line with the suggestions of some authors (e.g., de Lange et al., 2004; Boersma and Lindblom, 2009) who argued of taking into account in surveys on burnout an interval of at least 1 year between the study waves, a two-wave longitudinal study with a 14 months-time interval took place among nurses of an Italian academic hospital. The data collections were conducted in 2 months (March and May). During these months the hospitals are not particularly flooded with patients, as is the case in the months before and after summer, and the Christmas holidays. Finally, we checked whether important organizational changes (e.g., downsizing, re-organization) had taken place in the hospital. This was not the case in the 14 months interval between baseline and follow-up. We approached subjects during workshops of the in-service training curriculum, and provided information about the purpose and design of the study. The voluntary nature of the study was emphasized. Data were collected by means of paper and pencil questionnaires. Sixty four subjects did not attend the courses for several reasons (e.g., they were not interested, or they were not available) and/or were not available to participate to survey. For privacy reasons, personal data of this group were not available. At both measurement times, we asked the respondents to generate an anonymous code. By means of this code we were able to link the questionnaires at both points in time. In Italy, ethical approval from the ethics committee of participating hospitals is required, and approval was granted by the ethics committee of S. Maria Hospital, Terni, Italy.

The study population consisted of 287 nurses from an Italian hospital. All nurses worked on a permanent basis. At Time 1, 264 (92%) usable questionnaires were returned. At Time 2, 217 (drop out 47 = 19%) questionnaires were returned. Our final study sample consisted of these 217 nurses who filled out both questionnaires (response rate of 76% of the initial group). Of these respondents, the majority was female (84%). The mean age was 42.7 years (SD = 7.2; range = 28–56). On the average the respondents had been working in a health care setting for 17.0 years (SD = 9.1; range = 1–37). Participants who completed both questionnaires and those who only participated in the baseline survey did not differ significantly on any demographic variable (age, gender, education, number of cohabitating children), or psychosocial job characteristic (JDCS), or burnout dimension.

### Measures

#### Demographic variables

Age was measured in years and gender was categorized as 1 = male and 2 = female.

#### JDCS variables

These variables were measured with three scales of the Italian version of the Leiden Quality of Work Life Questionnaire for Nurses (LQWLQ-N; Maes et al., 1999; Pisanti, 2007; Pisanti et al., 2009). These three LQWQ-N scales provide an occupation-specific measurement corresponding closely to the original operationalization of job demands, control, and social support in the Job Content Instrument (JCI; Karasek, 1985). Responses are measured on a 4-point scale ranging from 1 (*totally disagree*) to 4 (*totally agree*). Job demands were measured with one scale (work and time pressure: 4 items; e.g., “I must care for too many patients at once”). Control was assessed using a composite scale of skill discretion (4 items; e.g., “My work is varied.”) and decision authority (4 items; e.g., “I can decide for myself when to carry out patient-related tasks and when to carry out non-patient-related tasks.”). According to Karasek and Theorell (1990) skill discretion and decision authority are theoretically and empirically closely related and therefore often combined in one scale. A composite measure is frequently used in research on the JDCS model, also in studies on nurses (e.g., Bourbonnais et al., 1998; Bakker et al., 2005). Perceived emotional and practical social support was assessed with two subscales: social support from supervisor (6 items; e.g., “I can count on the support of my direct supervisor when I face a problem at work.”) and social support from co-workers (6 items; e.g., “The nurses in my department work well together.”). Inspired by the papers of Bourbonnais et al. (1999) and Karasek (1985), both scales were integrated into one social support scale.

To examine the factorial structure of three scales of LQWLQ-N, we conducted principal component analysis (PCA) both at Time 1 and at Time 2. In both cases PCA revealed the presence of three factors explaining 50% of the variance at Time 1 and 56% of the variance at Time 2. In both cases an inspection of the scree plot revealed a clear break after the third component. These results were further supported by the results of parallel analysis, which in both measurements showed three dimensions with eigenvalues exceeding the corresponding criterion values for a randomly generated data matrix of the same size.

#### Burnout

*Burnout* was assessed by the Italian version (Pisanti et al., 2013) of the 20-item Maslach Burnout Inventory Human Service Survey (MBI-HSS; Maslach et al., 1996) which contains the three subscales: emotional exhaustion (8 items; e.g., “I feel emotionally drained from my work.”); depersonalisation (5 items; e.g., “I don't really care what happens to some patients”) and personal accomplishment (7 items; e.g., “I deal very effectively with the problems of my recipients.”). Participants were asked to rate from 0 (*never*) to 6 (*daily*) how often they experienced feelings described in each of the 20-items.

To analyze the factorial structure of the MBI-HSS, we carried out principal component analysis (PCA) both at Time 1 and at Time 2. In both cases PCA revealed the presence of three factors explaining 56% of the variance at Time 1 and 51% of the variance at Time 2. In both cases an inspection of the scree plot showed a clear break after the third component. These results were further supported by the results of parallel analysis, which in both measurements revealed three dimensions with eigenvalues exceeding the corresponding criterion values for a randomly generated data matrix of the same size.

### Data analysis

The hypotheses were tested in hierarchical regression analyses. Five blocks of variables were created (see **Table 2**).

In the first block we controlled for the variables gender and age. Moreover, we included in the first block the outcome measured at Time 1. The second block concerned the main effects of job demands, job control, and social support measured at Time 1. Subsequently, the two way (third block) and three way interactions (fourth block) between the JDCS variables measured at Time 1 were considered in the model. To avoid multi-collinearity and to facilitate the interpretation of the interaction terms, the scores on the job conditions were standardized before analysis (Cohen et al., 2003).

The second hypothesis focused on across-time changes in burnout as a function of the changes of JDCS dimensions across time. First, in line with Taris (2000) and Smith and Beaton (2008), a change score (the residual score derived by regressing each psychosocial job condition measured at Time 2 on the corresponding Time 1 score) was computed for each job condition. Next, in the final step of the hierarchical regression analyses, the change scores of each JDCS variable were entered.

Following the suggestions of Becker (2005) and Spector and Brannick (2011), we first checked the influence of the control variables gender and age on the final models. We ran the analysis with and without the control variables. Since the pattern of results was largely similar, we reported the findings of the more parsimonious model without control variables. Moreover, in line with the suggestions of Ford et al. (2014), we also considered the potential dynamics of reverse causation effects, thus we reversed the relationships of the hypothesized model psychosocial job dimensions T1 predicting Burnout variables T2, to test a reverse causation model, i.e., the Time 1 burnout dimensions explaining the Time 2 psychosocial job dimensions controlling for the psychosocial job dimensions at Time 1.

## Results

Descriptive data and zero-order Pearson correlations of the study variables are displayed in Table 1. All scales measuring the study variables displayed acceptable levels of reliability (alpha coefficients ranged from 0.67 to 0.96). Furthermore, Table 1 shows that the auto-correlations of the JCDS variables between Time 1 and Time 2 vary from 0.35 (Job Control) to 0.48 (Social Support), indicating small, or moderate levels of auto-correlation. This finding supports the above mentioned argument that the psychosocial job variables are not very stable over time. The same applies to the burnout variables, showing T1–T2 auto-correlations ranging from 0.32 (Personal Accomplishment) to 0.53 (Emotional Exhaustion).

**Table 1:** **Means (***M***), standard deviations (***SD***), internal consistencies (Cronbach's α), and zero-order correlations of the study variables (***N*** = 217)**.

**Variable**	***M***	***SD***	**α**	**1**	**2**	**3**	**4**	**5**	**6**	**7**	**8**	**9**	**10**	**11**	**12**	**13**
**BACKGROUND VARIABLES**																
(1) Gender^a^	–		–	–												
(2) Age	42.7	7.2	–	−0.10	–											
**TIME 1**																
(3) JD	2.8	0.6	0.71	0.09	−0.08	–										
(4) JC	2.7	0.5	0.80	−0.05	0.16^*^	−0.13	–									
(5) SS	2.7	0.6	0.86	−0.15^*^	0.10	−0.16^*^	0.39^***^	–								
(6) EE	2.5	1.3	0.89	0.20^**^	0.11	0.19^**^	−0.34^***^	−0.25^***^	–							
(7) DP	1.1	1.1	0.72	−0.18^**^	0.03	0.17^*^	−0.22^**^	−0.05	0.42^***^	–						
(8) PA	4.4	1.1	0.87	0.03	0.05	−0.02	0.24^***^	0.01	−0.16^*^	−0.41^***^	–					
**TIME 2**																
(9) JD	2.7	0.6	0.79	0.16^*^	−0.04	0.37^***^	−0.22^***^	−0.09	0.17^*^	0.04	−0.05	–				
(10) JC	2.7	0.4	0.76	−0.05	−0.03	0.06	0.35^***^	0.37^***^	−0.19^**^	−0.08	0.10	−0.13	–			
(11) SS	2.7	0.5	0.88	−0.03	−0.03	−0.06	0.23^***^	0.48^***^	−0.15^*^	0.01	0.08	−0.16^*^	0.45^***^	–		
(12) EE	2.8	0.9	0.83	0.24^***^	0.20^**^	0.08	−0.18^*^	0.02	0.53^***^	0.18^*^	−0.20^**^	0.25^***^	−0.33^***^	−0.30^***^	–	
(13) DP	1.1	0.8	0.67	−0.22^***^	−0.11	−0.01	0.08	0.12	0.11	0.36^***^	−0.28^***^	0.25^***^	−0.07	0.03	0.17^*^	–
(14) PA	4.1	0.7	0.76	0.17^*^	0.04	−0.17^*^	0.32^***^	0.16^*^	−0.14^*^	−0.25^***^	0.32^***^	−0.13	0.29^***^	0.19^**^	−0.12	−0.36^***^

a *Male, 1; Female, 2*.

* *p < 0.05*;

** *p < 0.01*;

*** *p < 0.001*.

*JD, Job demands; JC, Job control; SS, Social support; EE, Emotional exhaustion; DP, Depersonalization; PA, Personal accomplishment*.

### Testing the additive and interactive effects of the JDCS model

Regarding emotional exhaustion, the results in Table 2 show that Time 1 emotional exhaustion (Beta = 0.49, *p* < 0.001) accounted for 24% of the variance in Time 2 emotional exhaustion [*F*_*change*(1, 183)_ = 58.8, *p* < 0.001]. In the second block, the inclusion of the main effects of Time 1 JDCS variables did not significantly improve the prediction of Time 2 emotional exhaustion [2% of explained variance, *F*_*change*(3, 180)_ = 1.6, *p* > 0.05] (Hypothesis 1a was not supported). However social support measured at Time 1 revealed a significant association (Beta = 0.14, *p* < 0.05) with emotional exhaustion measured at Time 2. In contrast to our assumption, social support had a positive effect on emotional exhaustion. Inclusion of the Time 1 JDCS two-way (Block 3) and three-way interactions (Block 4) did not significantly improve the prediction of Time 2 emotional exhaustion (Hypothesis 1b was not supported).

**Table 2:** **Results of hierarchical regression analyses examining the effect of the burnout dimension measured at time 1, psychosocial job dimensions measured at time 1, and changes in psychosocial job dimensions between Time 1 and Time 2 on the three burnout dimensions assessed at Time 2 (***N*** = 217)**.

**Variables**	**Emotional exhaustion at time 2**	**Depersonalization at time 2**	**Personal accomplishment at time 2**
Burnout dimension T1	0.49^***^	0.48^***^	0.41^***^
**Block 1** Δ***R***^2^	0.24^***^	0.21^***^	0.17^***^
Burnout dimension T1	0.49^***^	0.47^***^	0.32^***^
Demands T1	−0.01	−0.08	0.23^***^
Control T1	−0.11	0.07	0.22^**^
Social support T1	0.14^*^	0.17^*^	0.06
**Block 2** Δ***R***^2^ **(*****R***^2^**)**	0.02 (26)	0.02^*^ (0.23)	0.08^***^(25)
Burnout dimension T1	0.51^***^	0.46^***^	0.32^***^
Demands T1	−0.04	−0.09	0.22^**^
Control T1	−0.10	0.08	0.21^**^
Social support T1	0.14^*^	0.16^*^	0.06
Demands T1 × Control T1	0.12	0.09	0.00
Demands T1 × Social support T1	−0.12	−0.11	0.08
Control T1 × Social support T1	−0.08	0.00	−0.02
**Block 3** Δ***R***^2^ **(*****R***^2^**)**	0.03* (29)	0.01 (0.24)	0.01 (26)
Burnout dimension T1	0.53^***^	0.47^***^	0.33^***^
Demands T1	−0.05	−0.10	0.21^**^
Control T1	−0.07	0.10	0.27^***^
Social support T1	0.15^*^	0.16^*^	0.09
Demands T1 × Control T1	0.06	0.04	−0.08
Demands T1 × Social support T1	−0.11	−0.10	0.09
Control T1 × Social support T1	−0.03	0.05	0.06
Demands T1 × Control T1 × Social support T1	0.13	0.12	0.20^*^
**Block 4** Δ***R***^2^ **(*****R***^2^**)**	0.01 (30)	0.00 (0.24)	0.02^*^(0.28)
Burnout dimension T1	0.48^***^	0.47^***^	0.32^***^
Demands T1	−0.04	0.09	0.20^**^
Control T1	−0.08	0.08	0.25^**^
Social support T1	0.22^***^	0.15^*^	0.07
Demands T1 × Control T1	0.12	0.01	−0.08
Demands T1 × Social support T1	−0.07	−0.09	0.08
Control T1 × Social support T1	−0.04	0.06	0.06
Demands T1 × Control T1 × Social support T1	0.12	0.14	0.20^*^
Δ Demands	0.13^*^	0.32^***^	−0.14^*^
Δ Control	−0.29^***^	−0.13	0.10
Δ Social support	−0.22^***^	0.06	0.02
**Block 5** Δ***R***^2^**(*****R***^2^**)**	0.20^***^(50)	0.11^***^ **(0.35)**	0.03^*^ (0.31)
***R***^2^	**50**	0.35^***^	**0.33**
**Adj** ***R***	**0.48**	**0.31**	**0.30**
**Full Model**	***F*_(11, 184)_ = 12.59**	***F*_(11, 187)_ = 5.99**	***F*_(11, 187)_ = 7.30**

The standardized regression coefficients (Beta's) are reported, the bold values are significant at

* *p < 0.05*;

** *p < 0.01*;

*** *p < 0.001*;

*Block n DR^2^, R Square Change*. Δ *Demands*, Δ *Control*, Δ *Social Support: changes scores in Demands, Control, and Social support*.

As concerns depersonalization, the first block [*F*_*change*__(1, 186)_ = 39.5, *p* < 0.001, Δ*R*^2^ = 21%] and the second block [*F*_*change*(3, 183)_ = 3.9, *p* < 0.05, Δ*R*^2^ = 2%] accounted for significant variance in Time 2 depersonalization. Time 1 depersonalization was the most important predictor by far (Beta = 0.48, *p* < 0.001). In the second block, social support measured at Time 1 showed a positive association (Beta = 0.17, *p* < 0.05) with depersonalization measured at Time 2. (Hypothesis 1a was not supported). Also in this case, inclusion of the Time 1 JDCS two-way (Block 3) and three-way interactions (Block 4) did not significantly improve the prediction of Time 2 depersonalization (Hypothesis 1b was not supported).

**Figure 1:**
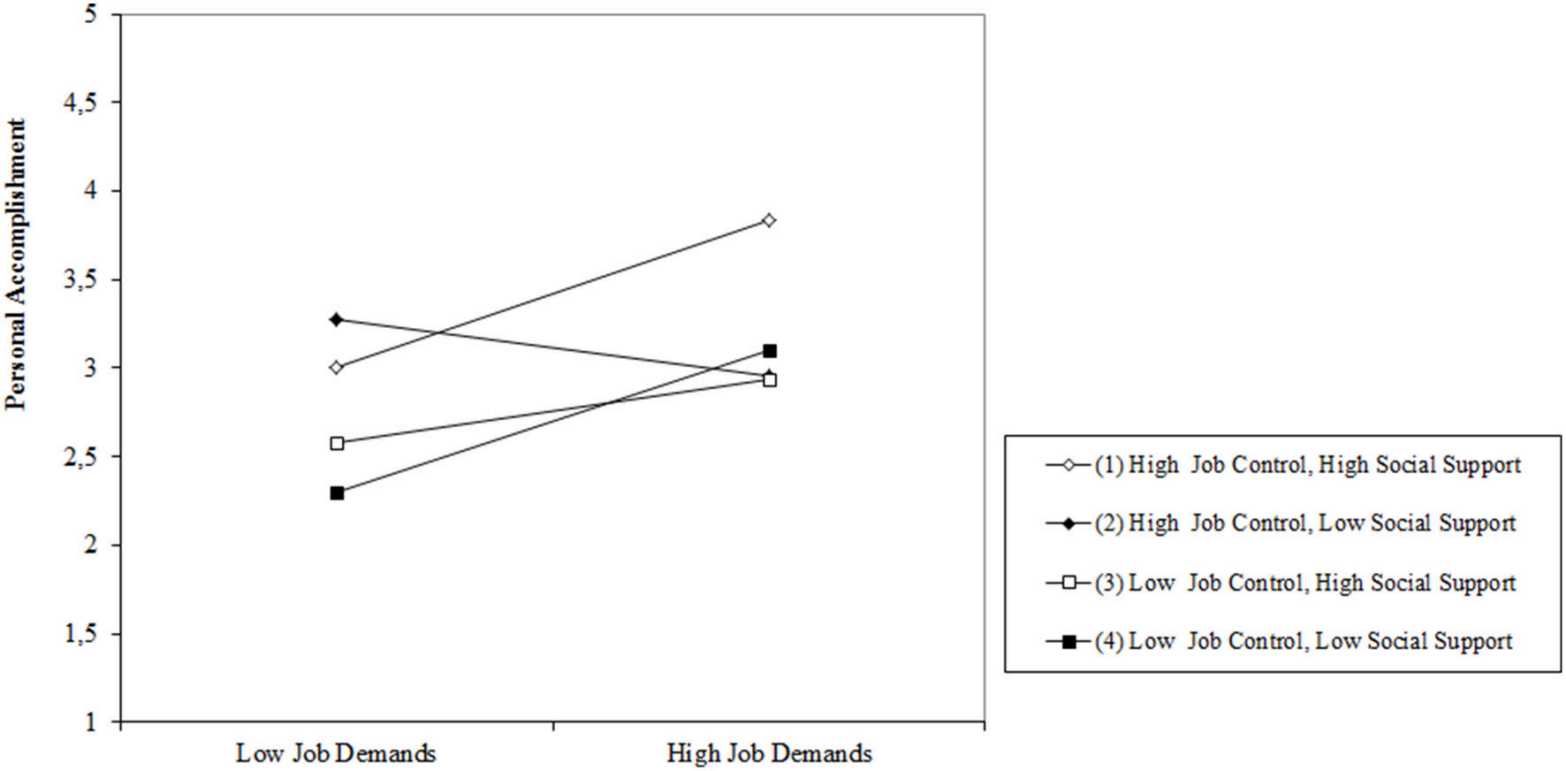
**Job demands × job control × social support, predicting personal accomplishment**.

Finally, personal accomplishment was mainly predicted by variables included in the Block 1, Block 2, and Block 4. The effect of Time 1 personal accomplishment (Beta = 0.41, *p* < 0.001) accounted for a significant proportion of the variance in the outcome variable [*F*_*change*(1, 186)_ = 37.1, *p* < 0.001, Δ*R*^2^ = 17%]. Inclusion of the main effects of Time 1 JDCS variables improved the prediction of Time 2 personal accomplishment significantly by 8% [*F*_*change*(3, 183)_ = 6.8, *p* < 0.001, Δ*R*^2^ = 8%]. Both Time 1 job demands (Beta = 0.23, *p* < 0.001) and Time 1 job control (Beta = 0.22, *p* < 0.01) were associated with Time 2 personal accomplishment. High job demands and high job control at Time 1 were associated with high personal accomplishment at Time 2 (Hypothesis 1a was not supported). Inclusion of the Time 1 two-way interactive effects (Model 3) did not significantly improve the prediction of Time 2 personal accomplishment. In block 4 the three-way interactive term demands × control × social support measured at Time 1 accounted for a significant additional proportion of variance [*F*_*change*(1, 179)_ = 6.5, *p* < 0.001, Δ*R*^2^ = 3%]. The significant interaction effect was graphically represented according to the method described by Cohen et al. (2003). Figure 1 shows that the interaction pattern (Beta = 0.20, *p* < 0.05) was synergistic or enhancing (Cohen et al., 2003): The highest sense of personal accomplishment was observed for nurses who perceived high demands, high control, and high social support. No support was found for a combined buffering effect of job control and social support on job demands (Hypothesis 1b was not supported).

All effects of the reverse causation models were non-significant, indicating that there was no evidence for any reverse causation.

### Testing associations between changes in JDCS dimensions and changes in burnout

Regarding our second hypothesis, the analyses in Table 2 (Block 5) indicated to what extent changes in psychosocial job variables are associated with changes in the burnout outcomes. Controlling for the initial burnout dimension and for baseline job characteristics, the blocks that included the changes in job conditions explained significant additional variance in all outcomes measured at Time 2. The change scores explained an additional 20% of the variance in emotional exhaustion at Time 2 [*F*_*change*(3, 173)_ = 23.9, *p* < 0.001]. Those employees who showed an increase in job demands (Beta = 0.13, *p* < 0.05) and a decrease in job control (Beta = −0.29, *p* < 0.001) and social support (Beta = −0.22, *p* < 0.005) over time, reported more emotional exhaustion at Time 2 (Hypothesis 2 was supported). With regard to depersonalization, the block with change scores accounted for an additional 10% of variance [*F*_*change*(3, 176)_ = 8.9, *p* < 0.001). The results showed that an increase in job demands (Beta = 0.30, *p* < 0.001) was associated with an increase of depersonalization over time (Hypothesis 2 was only supported for demands). Regarding personal accomplishment, again the changes in job conditions between T1 and T2 contributed significantly to the (change in) personal accomplishment at T2. The block including the change scores accounted for an additional 3% of the variance [*F*_*change*(3, 176)_ = 2.5, *p* < 0.05): a decrease in job demands (Beta = −0.14, *p* < 0.05) was related to an increase in personal accomplishment over time. Also in this case Hypothesis 2 was only supported for demands: across-time changes in job demands were associated with changes in personal accomplishment over time.

## Discussion

Firstly, we hypothesized that high demands, low control, and low social support (all measured at Time 1) would longitudinally contribute to high burnout (high emotional exhaustion, depersonalization, and low personal accomplishment, all measured 14 months later—Time 2). In line with previous longitudinal studies (e.g., Gelsema et al., 2006; Adriaenssens et al., 2013) our results did not support *Hypothesis 1a*. Probably the influence of psychosocial job dimensions have on each burnout variable is already accounted for by the inclusion of the baseline burnout score. The three burnout dimensions appeared differentially related to the hypothesized cross lagged main effects of demands, control, and social support.

Concerning the core dimensions of burnout (emotional exhaustion and depersonalization; Breso et al., 2007) the results showed that the employees, who experienced high levels of social support at Time 1, revealed higher levels of emotional exhaustion and depersonalization at Time 2. These results were not in line with our hypothesis. However, several studies have shown that under certain circumstances high levels of social support could have negative effects (de Jonge and Schaufeli, 1998; Wallace, 2005; Johnston et al., 2013). In line with the stress transfer theory (Karasek et al., 1982) less strained people could assimilate the strain of colleagues more stressed. In other words, in situations with strong social bonds, individuals may absorb more feelings of stress from those around them rather than be protected from stress (Karasek et al., 1982; Wallace, 2005; Johnston et al., 2013).

We should note that among the JDCS variables, contrary to our hypothesis and literature mentioned in the introduction (Lee and Ashforth, 1996; Schaufeli, 2007), job demands did not show a significant cross lagged association with emotional exhaustion and depersonalization. This result can be explained on the basis of the findings of Teuchmann et al. (1999). They found that job demands (operationalized in terms of time pressure as in the present study) fluctuated in parallel with emotional exhaustion over time. Likewise, in the present study we found significant cross sectional associations between job demands and emotional exhaustion both at Time 1 and at Time 2, suggesting that these two dimensions fluctuate concomitantly over time.

Finally, with respect to personal accomplishment, we found that, after controlling for personal accomplishment measured at Time 1, high levels of job demands and control (measured at Time 1) were significantly cross lagged related with high levels of personal accomplishment measured at Time 2. These findings do not support our Hypothesis 1a: only the relationship between job control and personal accomplishment was in line with our predictions. However, the positive association between high levels of demands and personal accomplishment has been previously reported (van Vegchel et al., 2004; Lee and Akhtar, 2007), and can be explained as follows. When high job control occurs in conjunction with high job demands (“active job”), it is hypothesized that employees are able to deal with these demands, protecting them from excessive strain, fostering in them feelings of learning and of mastery, and leading them to positive states, such as motivation and personal accomplishment (Karasek and Theorell, 1990).

Beyond the main effects previously discussed, we found a small but significant three-way interaction effect between job demands, job control, and social support on personal accomplishment. However, the pattern of conditional relationships was not consistent with our hypothesis (moderating pattern). The strength of the relationship between job demands measured at Time 1 and personal accomplishment measured at Time 2 increased as the values of job control and social support (both measured at time 1) increased. Thus the pattern was synergistic. This means that nurses, who perceived high job demands and high job control (previously described “active jobs”) in conjunction with high social support at Time 1, experienced more personal accomplishment at Time 2, then their colleagues who experienced high job demands, high job control, and low social support at time 1 (simple slope test: *t* = 2.76, *p* < 0.01).

Although we adopted a specific measurement of the JDCS variables for nurses, hypothesis 2b was not supported in our study. This finding is in line with Taris (2006), who concluded that full support for the buffer hypothesis was found in a small percentage of studies, little more than chance level. The available evidence suggests that the moderating effect is an exception rather than the rule. The inconsistencies in demonstrating interaction effects between job demands and job resources may also be due to a lack of match between the kind of occupational stressors examined in combination with a specific form of job resource (job control and/or social support). de Jonge and Dormann (2006) argued that stressors and resources need to address similar domains of functioning (i.e., cognitive, emotional, physical) in order to interact in the prediction of domain specific strains.

Inclusion of changes in job conditions (Δ Demands, Δ Control, and Δ Social support) improved the prediction of burnout dimensions (3–20% of additional explained variance). Differential patterns of relationships between (changes in) job conditions and (changes in) burnout dimensions were found. In accordance with other longitudinal studies conducted among nurses in other countries (Bourbonnais et al., 1999; Burisch, 2002; Gelsema et al., 2006), changes in emotional exhaustion were most strongly influenced by increases in job demands and decreases in both job control and social support. This final model explained 50% of the variance in emotional exhaustion. With respect to depersonalization, the full model explained 35% of variance and the only significant predictor was changes in job demands: more specifically increases in job demands were associated with increases in depersonalization at Time 2. Finally, as regards personal accomplishment, the full model explained 33% of the variance. Decreases in job demands across time were associated with higher levels of Time 2 personal accomplishment. Furthermore, we can see a seemingly contradictory effect of job demands and change in demands on personal accomplishment. However, as suggested in a recent paper by Warr and Inceoglu (2012), high levels of job demands and challenges can be attractive for employees with higher levels of personal accomplishment and higher engagement in a job, “…leading them to become drawn in and still more energized.” (page 131). Over time, increases of job demands could be deleterious leading employees to drain their feelings of personal accomplishment. Overall, this pattern of results suggests that an increase over time of job demands tends to result in elevated levels of all dimensions of burnout over time (high emotional exhaustion, depersonalization, and low personal accomplishment); however, only for emotional exhaustion the changes in job control and in social support seem to have a detrimental effect. This finding is in line with the general literature on burnout (Schaufeli, 2007): among all burnout dimensions, emotional exhaustion seems most strongly influenced by the psychosocial job conditions.

### Strengths and limitations

A first strength of the present study is that we tested our hypotheses in a two-wave panel research. Secondly, we focused on the effects of changes in the JDCS variables on (changes) in burnout. Longitudinal studies in this specific area appear to be rather scarce (e.g., Taris and Kompier, 2003), and have seldom investigated the influence of changes in psychosocial job characteristics on (changes in) burnout levels. Overall, our findings are in line with previous studies (Boersma and Lindblom, 2009; Schaufeli et al., 2011) which have shown how psychosocial job dimensions and burnout variables can be explained by a component reflecting stability and a component reflecting change in these constructs. Moreover, our results support the validity of the theoretical models postulating a causal link between changes in psychosocial job characteristics and (changes in) burnout dimensions. These results also suggest that improvements in psychosocial job variables through organizational interventions can have positive effects on nurse's burnout. Finally, our findings about the reverse causation effects are largely consistent with a recent quantitative review (Tang, 2014). The author found a weak evidence in support of a positive strain (emotional exhaustion and depersonalization)-to-job demands effect, but he did not find any support for either a strain-to-job control or for a strain-to-workplace social support effect.

Two limitations of the study should be noted. Firstly, the current data set was drawn from a specific group of employees (nurses, all working for the same organization). Organizational macro processes (Giorgi et al., 2015) such as culture (Schneider et al., 2013) and climate (Giorgi, 2012; Schneider et al., 2013) dimensions may affect both psychosocial job dimensions and burnout variables. However, de Lange et al. (2003) have concluded in their review that studies based on heterogeneous populations do not provide more support for the JDCS hypotheses than studies based on homogeneous samples “.this suggests that homogeneous populations provide enough true individual and within-occupation variation in job characteristics (i.e., provide enough exposure contrast) to be as useful as heterogeneous samples in testing the DCS model.” (de Lange et al., 2003; page 300). Nevertheless, the specific nature of the present sample underlines the need to replicate the current findings on different occupational groups. Secondly, although two-wave longitudinal designs offer better opportunities for testing cross lagged associations than cross sectional studies, a more comprehensive examination of the cross-lagged relations between psychosocial job variables and burnout would require a multi-wave study to get more insight into the process regarding the impact of changes of psychosocial job characteristics on (changes in) burnout dimensions (Taris and Kompier, 2003).

In agreement with these reservations, it seems important that future longitudinal multi waves research analyzes the hypotheses presented in this study in different occupational groups.

### Implications

The present study found evidence for longitudinal relationships between JDCS variables and occupational burnout. The results are encouraging because they suggest that job redesign interventions, focusing on improvement of psychosocial job characteristics may be an effective tool to prevent and reduce burnout.

According to Schalk et al. (2010), these improvements could be achieved by organizational interventions such as changing routines/responsibilities, organizing team meetings, and training in leadership qualities for supervisors (providing feedback and support, coaching). These strategies may augment nurse's job resources such as job control and social support. Moreover taking into account the positive cross lagged associations between social support and the core dimensions of burnout (emotional exhaustion and depersonalization), occupational health psychologists should pay attention to optimize the quality of teambuilding inside the nurses teams. These interventions should be integrated into current management activities. We should bear in mind that these intervention strategies are more effective if they are permanent rather than temporary and occasional: managing work-related stress is not a one-off activity but part of a continuing cycle of good management at work and of the effective management of occupational stress and well-being.

In conclusion, our study underlines the importance of investigating the associations between the changes in psychosocial job variables and the (changes in) burnout dimensions, across time. Even after controlling for demographic variables, burnout, and psychosocial job characteristics at Time 1, the effects of changes in psychosocial job variables on changes in burnout dimensions remained of interest. Thus, it appears that in future research more attention for this phenomenon is warranted, and also across time development in psychosocial job variables should be examined rather than focusing solely on their “static” effects. From a practical point of view, these findings suggest that interventions to promote favorable psychosocial changes may positively influence employees' levels of burnout. A next step would be to conduct experimental studies (Le Blanc et al., 2007) to examine whether through favorable changes in psychosocial job characteristics, burnout can be prevented or reduced.

## Author contributions

RP conceived and designed this study, collected, and analyzed the data and wrote the paper. MV contributed to writing the paper. SM, LM, DL, and CV were involved in data acquisition. RP, MD, and LM, were involved in the statistical analyses for the project. RP, MV, LM, SM, DL, and CV agree to be accountable for all aspects of the work specifically to responding to questions related to the accuracy or integrity of any part of the work.

### Conflict of interest statement

The authors declare that the research was conducted in the absence of any commercial or financial relationships that could be construed as a potential conflict of interest.

## References

[B1] Adriaenssens J. de Gucht V. Maes S. (2013). Causes and consequences of occupational stress in emergency nurses, a longitudinal study. J. Nurs. Manag. 57, 151–160. 10.1111/jonm.12138 24330154

[B2] Bakker A. B. Le Blanc P. M. Schaufeli W. B. (2005). Burnout contagion among intensive care nurses. J. Adv. Nurs. 51, 276–287. 10.1111/j.1365-2648.2005.03494.x 16033595

[B3] Becker T. E. (2005). Potential problems in the statistical control of variables in organizational research: a qualitative analysis with recommendations. Organ. Res. Methods 8, 274–289. 10.1177/1094428105278021

[B4] Boersma K. Lindblom K. (2009). Stability and change in burnout profiles over time: a prospective study in the working population. Work Stress 23, 264–283. 10.1080/02678370903265860

[B5] Bourbonnais R. Comeau M. Vezina M. (1999). Job strain and evolution of mental health among nurses. J. Occup. Health Psychol. 4, 95–107. 10.1037/1076-8998.4.2.95 10212863

[B6] Bourbonnais R. Comeau M. Vézina M. Dion G. (1998). Job strain, psychological distress, and burnout in nurses. Am. J. Ind. Med. 34, 20–28. 10.1002/(SICI)1097-0274(199807)34:1<20::AID-AJIM4>3.0.CO;2-U 9617384

[B7] Breso E. Salanova M. Schaufeli W. B. (2007). In search of the “third dimension” of burnout: efficacy or inefficacy? Appl. Psychol. Int. Rev. 56, 460–478. 10.1111/j.1464-0597.2007.00290.x26688003

[B8] Burisch M. (2002). A longitudinal study of burnout: the relative importance of dispositions and experiences. Work Stress 16, 1–17. 10.1080/02678370110112506

[B9] Canadas-De la Fuente G. A. Vargas C. San Luis C. Garcia I. Canadas G. R. De la Fuente E. I. (2015). Risk factors and prevalence of burnout syndrome in the nursing profession. Int. J. Nurs. Stud. 52, 240–249. 10.1016/j.ijnurstu.2014.07.001 25062805

[B10] Cohen J. Cohen P. West S. Aiken L. (2003). Applied Multiple Regression/Correlation Analysis for the Behavioral Sciences, 3rd Edn. Mahwah, NJ: Lawrence Erlbaum Associates Publishers.

[B11] Converso D. Loera B. Viotti S. Martini M. (2015). Do positive relations with patients play a protective role for healthcare employees? Effects of patients' gratitude and support on nurses' burnout. Front. Psychol. 6:470. 10.3389/fpsyg.2015.00470 25954227 PMC4404725

[B12] Cortese C. G. Colombo L. Ghislieri C. (2010). Determinants of nurses' job satisfaction: the role of work–family conflict, job demand, emotional charge and social support. J. Nurs. Manag. 18, 35–43. 10.1111/j.1365-2834.2009.01064.x 20465727

[B13] de Jonge J. Dormann C. (2006). Stressors, resources, and strain at work: a longitudinal test of the triple match principle. J. Appl. Psychol. 91, 1359–1374. 10.1037/0021-9010.91.5.1359 17100490

[B14] de Jonge J. Schaufeli W. B. (1998). Job characteristics and employee well-being: a test of Warr's Vitamin Model in health care workers using structural equation modeling. J. Organ. Behav. 19, 387–407. 10.1002/(SICI)1099-1379(199807)19:4<387::AID-JOB851>3.0.CO;2-9

[B15] de Jonge J. van Breukelen G. J. P. Landeweerd J. A. Nijhuis F. J. N. (1999). Comparing group and individual level assessments of job characteristics in testing the job demand-control model: a multilevel approach. Hum. Relat. 52, 95–122. 10.1177/001872679905200106

[B16] de Lange A. H. Taris T. W. Kompier M. A. J. Houtman I. L. D. Bongers P. M. (2002). Effects of stable and changing demand-control histories on worker health. Scand. J. Work Environ. Health 28, 94–108. 10.5271/sjweh.653 12019593

[B17] de Lange A. H. Taris T. W. Kompier M. A. J. Houtman I. L. D. Bongers P. M. (2003). The very best of the millennium: longitudinal research and the demand-control (-support) model. J. Occup. Health Psychol. 8, 282–305. 10.1037/1076-8998.8.4.282 14570524

[B18] de Lange A. H. Taris T. W. Kompier M. A. J. Houtman I. L. D. Bongers P. M. (2004). The relationships between work characteristics and mental health: examining normal, reversed and reciprocal relationships in a 4-wave study. Work Stress 18, 149–166. 10.1080/02678370412331270860

[B19] De Rijk A. E. Le Blanc P. M. Schaufeli W. B. de Jonge J. (1998). Active coping and need for control as moderators of the job demand-control model: effects on burnout. J. Occup. Organ. Psychol. 71, 1–18.

[B20] Ford M. T. Matthews R. A. Wooldridge J. D. Mishra V. Kakar U. M. Strahan S. R. (2014). How do occupational stressor-strain effects vary with time? A review and meta-analysis of the relevance of time lags in longitudinal studies. Work Stress 28, 9–30. 10.1080/02678373.2013.877096

[B21] Ganster D. C. Fox M. L. Dwyer D. J. (2001). Explaining employees' health care costs: a prospective examination of stressful job demands, personal control, and physiological reactivity. J. Appl. Psychol. 86, 954–964. 10.1037/0021-9010.86.5.954 11596811

[B22] Gelsema T. I. van der Doef M. Maes S. Janssen M. Akerboom S. Verhoeven C. (2006). A longitudinal study of job stress in the nursing profession: causes and consequences. J. Nurs. Manag. 14, 289–299. 10.1111/j.1365-2934.2006.00635.x 16629843

[B23] Giorgi G. (2012). Workplace bullying in academia creates a negative working environment. An Italian study. Employee Responsib. Rights J. 24, 261–275. 10.1007/s10672-012-9193-7

[B24] Giorgi G. Mancuso S. Fiz Perez F. Castiello D'Antonio A. Mucci N. Cupelli V.. (2016). Bullying among nurses and its relationship with burnout and organizational climate. Int. J. Nurs. Pract. 22, 160–168. 10.1111/ijn.12376 25825025

[B25] Giorgi G. Shoss M. K. Leon-Perez J. M. (2015). Going beyond workplace stressors: economic crisis and perceived employability in relation to psychological distress and job dissatisfaction. Int. J. Stress Manag. 22, 137–158. 10.1037/a0038900

[B26] Häusser J. A. Mojzisch A. Niesel M. Schulz-Hardt S. (2010). Ten years on: a review of recent research on the Job Demand-Control (-Support) model and psychological well-being. Work Stress 24, 1–35. 10.1080/02678371003683747

[B27] Hochwalder J. (2006). An empirical exploration of the effect of personality on general and job-related mental ill health. Soc. Behav. Pers. 34, 1051–1070. 10.2224/sbp.2006.34.9.1051

[B28] Hochwalder J. (2007). The psychosocial work environment and burnout among Swedish registered and assistant nurses: the main, mediating, and moderating role of empowerment. Nurs. Health Sci. 9, 205–211. 10.1111/j.1442-2018.2007.00323.x 17688479

[B29] Johnston C. S. de Bruin G. P. Györkös C. Geldenhuys M. Massoudi K. Rossier J. (2013). Sense of coherence and job characteristics in predicting burnout in a South African sample. SA J. Ind. Psychol. 39, 9 10.4102/sajip.v39i1.1096

[B30] Johnson J. V. Hall E. (1988). Job strain, workplace social support and cardiovascular disease: a cross sectional study of a random sample of the Swedish working population. Am. J. Public Health 78, 1336–1342. 10.2105/AJPH.78.10.1336 3421392 PMC1349434

[B31] Karasek R. A. (1985). Job Content Questionnaire and User's Guide (Revision 1.1). Lowell, MA: University of Massachusetts Lowell, the Job Content Questionnaire Center.

[B32] Karasek R. A. Theorell T. (1990). Healthy Work, Stress, Productivity, and the Reconstruction of Working Life. New York, NY: Basic Books.

[B33] Karasek R. A. Triantis K. P. Chaudry S. S. (1982). Coworker and supervisor support as moderators of associations between task characteristics and mental strain. J. Occup. Behav. 3, 181–200. 10.1002/job.4030030205

[B34] Kasl S. V. (1996). The influence of the work environment on cardiovascular health: a historical, conceptual, and methodological perspective. J. Occup. Health Psychol. 1, 42–56. 10.1037/1076-8998.1.1.42 9547033

[B35] Kilfedder C. J. Power K. G. Wells T. J. (2001). Burnout in psychiatric nursing. J. Adv. Nurs. 34, 383–396. 10.1046/j.1365-2648.2001.01769.x 11328444

[B36] Landsbergis P. A. (1988). Occupational stress among health care workers: a test of the job demands-control model. J. Organ. Behav. 9, 217–239. 10.1002/job.4030090303

[B37] Le Blanc P. M. Hox J. J. Schaufeli W. B. Taris T. W. Peeters C. W. (2007). Take Care! the evaluation of a team-based burnout intervention program for oncology care providers. J. Appl. Psychol. 92, 213–227. 10.1037/0021-9010.92.1.213 17227163

[B38] Lee J. S. Y. Akhtar S. (2007). Job burnout among nurses in Hong Kong: implications for human resource practices and interventions. Asia Pac. J. Hum. Resour. 45, 63–84. 10.1177/1038411107073604

[B39] Lee R. T. Ashforth B. E. (1996). A meta-analytic examination of the correlates of three dimensions of job burnout. J. Appl. Psychol. 81, 123–133. 10.1037/0021-9010.81.2.123 8603909

[B40] Maes S. Akerboom S. van der Doef M. Verhoeven C. (1999). De Leidse Arbeids Kwaliteits Schaal voor Verpleegkundigen (LAKS-V). (The Leiden Quality of Work Life Questionnaire for Nurses (LQWLQ-nurse- s)). Leiden: Health Psychology, Leiden University.

[B41] Maslach C. Jackson S. Leiter M. P. (1996). Maslach Burnout Inventory Manual, 3rd Edn. Palo Alto, CA: Consulting Psychologists Press.

[B42] Maslach C. Schaufeli W. B. Leiter M. P. (2001). Job burnout. Annu. Rev. Psychol. 52, 397–422. 10.1146/annurev.psych.52.1.397 11148311

[B43] McVicar A. (2003). Workplace stress in nursing: a literature review. J. Adv. Nurs. 44, 633–642. 10.1046/j.0309-2402.2003.02853.x 14651686

[B44] Melamed S. Armon G. Shirom A. Shapira I. (2011). Exploring the reciprocal causal relationship between job strain and burnout: a longitudinal study of apparently healthy employed persons. Stress Health 27, 272–281. 10.1002/smi.1356

[B45] Narayanan L. Menon S. Spector P. E. (1999). Stress in the workplace: a comparison of gender and occupations. J. Organ. Behav. 20, 63–73. 10.1002/(SICI)1099-1379(199901)20:1<63::AID-JOB873>3.0.CO;2-J

[B46] Panagopoulou E. Montgomery A. J. Tsiga E. (2015). Bringing the well-being and patient safety research agenda together: why healthy HPs equal safe patients. Front. Psychol. 6:211. 10.3389/fpsyg.2015.00211 25774146 PMC4342865

[B47] Pisanti R. (2007). Una verifieca empirica del modello Domanda-Controllo-Sostegno Sociale: effetti sul burnout e sulla somatizzazione tra il personale infermieristico. [An empirical investigation of the demand-control-social support model: effects on burnout and on somatic complaints among nursing staff]. G. Ital. Med. Lav. Ergon. 29(1 SUPPL. A), 30–36. 17650740

[B49] Pisanti R. Lombardo C. Lucidi F. Violani C. Lazzari D. (2013). Psychometric properties of the Maslach Burnout inventory for human services among Italian nurses: a test of alternative models. J. Adv. Nurs. 69, 697–707. 10.1111/j.1365-2648.2012.06114.x 22897490

[B48] Pisanti R. van der Doef M. Maes S. Lazzari D. Bertini M. (2011). Job characteristics, organizational conditions, and distress/well-being among Italian and Dutch nurses: A cross-national comparison. Int. J. Nurs. Stud. 48, 829–837. 10.1016/j.ijnurstu.2010.12.006 21257172

[B50] Pisanti R. van der Doef M. Maes S. (2012). The job demand control (-Support) model and psychological well-being in nurses: a review, in Beyond the Job Demand Control (-Support) Model: Explaining Stress Reactions in Nurses, ed Pisanti R. (Leiden: Leiden University), 23–72.

[B51] Pisanti R. van der Doef M. Maes S. Lazzari D. Violani C. (2009). Psychometric properties of the Italian version of Leiden Quality of Work Questionnaire for Nurses (LQoWQ-N). Psychol. Health 24, 318.

[B52] Pisanti R. van der Doef M. Maes S. Lombardo C. Lazzari D. Violani C. (2015). Occupational coping self-efficacy explains distress and well-being in nurses beyond psychosocial job characteristics. Front. Psychol. 6:1143. 10.3389/fpsyg.2015.01143 26300827 PMC4526791

[B53] Proost K. de Witte H. de Witte K. Evers G. (2004). Burnout among nurses: extending the job demand-control-support model with work-home interference. Psychol. Belg. 44, 269–288. 10.5334/pb-44-4-269

[B54] Renzi C. Di Pietro C. Tabolli S. (2012). Psychiatric morbidity and emotional exhaustion among hospital physicians and nurses: association with perceived job-related factors. Arch. Environ. Occup. Health 67, 117–123. 10.1080/19338244.2011.578682 22524653

[B55] Roe R. (2008). Time in applied psychology: the study of “what happens” rather than “what is.” Eur. Psychol. 13, 37–52. 10.1027/1016-9040.13.1.37

[B56] Rudman A. Gustavsson J. P. (2012). Burnout during nursing education predicts lower occupational preparedness and future clinical performance: a longitudinal study. Int. J. Nurs. Stud. 49, 988–1001. 10.1016/j.ijnurstu.2012.03.010 22542085

[B57] Schalk D. M. Bijl M. L. Halfens R. J. Hollands L. Cummings G. G. (2010). Interventions aimed at improving the nursing work environment: a systematic review. Implement. Sci. 5, 34–44. 10.1186/1748-5908-5-34 20423492 PMC2876995

[B58] Schaufeli W. B. (2007). Burnout in health care, in Handbook of Human Factors and Ergonomics in Health Care and Patient Safety, ed Carayon P. (Mahway, NJ: Lawrence Erlbaum), 217–232.

[B59] Schaufeli W. B. Bakker A. B. van Rhenen W. (2009). How changes in job demands and resources predict burnout, work engagement, and sickness absenteeism. J. Organ. Behav. 30, 893–917. 10.1002/job.595

[B60] Schaufeli W. B. Maassen G. H. Bakker A. B. Sixma H. J. (2011). Stability and change in burnout: a 10-year follow-up study among primary care physicians. J. Occup. Organ. Psychol. 84, 248–267. 10.1111/j.2044-8325.2010.02013.x

[B61] Schmidt K. H. Diestel S. (2010). Differential effects of decision latitude and control on the job demands–strain relationship: a cross-sectional survey study among elderly care nursing staff. Int. J. Nurs. Stud. 48, 307–317. 10.1016/j.ijnurstu.2010.04.003 20472236

[B62] Schneider B. Ehrhart M. G. Macey W. H. (2013). Organizational climate and culture. Annu. Rev. Psychol. 64, 361–388. 10.1146/annurev-psych-113011-143809 22856467

[B63] Smith P. Beaton D. (2008). Measuring change in psychosocial working conditions: methodological issues to consider when data are collected at baseline and one follow-up time point. Occup. Environ. Med. 65, 288–296. 10.1136/oem.2006.032144 18349161

[B64] Spector P. E. Brannick M. T. (2011). Methodological urban legends: the misuse of statistical control variables. Organ. Res. Methods 14, 287–305. 10.1177/1094428110369842

[B65] Tang K. (2014). A reciprocal interplay between psychosocial job stressors and worker well-being? A systematic review of the “reversed” effect. Scand. J. Work Environ. Health 40, 441–456. 10.5271/sjweh.3431 24756578

[B66] Taris T. (2000). A Primer in Longitudinal Data Analysis. Thousand Oaks, CA: Sage publications.

[B67] Taris T. Kompier M. (2003). Challenges of longitudinal designs in occupational health psychology. Scand. J. Work Environ. Health 29, 1–4. 10.5271/sjweh.697 12630429

[B68] Taris T. W. (2006). Bricks without clay: on urban myths in occupational health psychology. Work Stress 20, 99–104. 10.1080/02678370600893410

[B69] Teuchmann K. Totterdell P. Parker S. K. (1999). Rushed, unhappy, and drained: an experience sampling study of relations between time pressure, perceived control, mood, and emotional exhaustion in a group of accountants. J. Occup. Health Psychol. 4, 37–54. 10.1037/1076-8998.4.1.37 10100112

[B70] Tummers G. E. R. Landeweerd J. A. van Merode G. G. (2002). Work organization, work characteristics, and their psychological effects on nurses in the Netherlands. Int. J. Stress Manag. 9, 183–206. 10.1023/A:1015519815319

[B71] Van Bogaert P. Clarke S. Vermeyen K. Meulemans H. Van de Heyning P. (2009). Practice environments and their associations with nurse reported outcomes in Belgian hospitals: development and preliminary validation of a Dutch adaptation of the revised nursing work index. Int. J. Nurs. Stud. 46, 54–64. 10.1016/j.ijnurstu.2008.07.009 18789437 PMC2845973

[B72] Van Bogaert P. Van Heusden D. Timmermans O. Franck E. (2014). Nurse work engagement impacts job outcome and nurse-assessed quality of care: model testing with nurse practice environment and nurse work characteristics as predictors. Front. Psychol. 5:1261. 10.3389/fpsyg.2014.01261 25431563 PMC4230203

[B73] van der Doef M. Maes S. (1999). The job demand-control (-support) model and psychological well-being: a review of 20 years of empirical research. Work Stress 13, 87–114. 10.1080/026783799296084

[B74] van der Doef M. Maes S. (2002). Teacher-specific quality of work versus general quality of work assessment: a comparison of their validity regarding burnout, (psycho)somatic well-being and job satisfaction. Anxiety Stress Coping 15, 327–344. 10.1080/1061580021000056500

[B75] van Vegchel N. Jonge J. Söderfeldt M. Dormann C. Schaufeli W. (2004). Quantitative versus emotional demands among Swedish human service employees: moderating effects of job control and social support. Int. J. Stress Manag. 11, 21–40. 10.1037/1072-5245.11.1.21

[B76] Wallace J. E. (2005). Job stress, depression and work-to-family conflict: a test of the strain and buffer hypotheses. Relat. Ind. 60, 510–539. 10.7202/012157ar

[B77] Warr P. Inceoglu I. (2012). Job engagement, job satisfaction, and contrasting associations with person–job fit. J. Occup. Health Psychol. 17, 129–138. 10.1037/a0026859 22308964

[B78] Welp A. Meier L. L. Manser T. (2015). Emotional exhaustion and workload predict clinician-rated and objective patient safety. Front. Psychol. 5:1573. 10.3389/fpsyg.2014.01573 25657627 PMC4302790

